# Nonfluoroscopic Ablation in the Setting of Congenital Heart Disease

**DOI:** 10.19102/icrm.2018.091005

**Published:** 2018-10-15

**Authors:** Vincent C. Thomas, Bhavya Trivedi

**Affiliations:** ^1^Department of Pediatrics, Pediatric Cardiology, University of Nebraska Medical Center, Omaha, NE, USA; ^2^Department of Pediatrics, Pediatric Cardiology, Florida Hospital for Children, Orlando, FL, USA

**Keywords:** Ablation, congenital heart disease, nonfluoroscopic, pediatrics

## Abstract

In this case series, we demonstrate the performance of nonfluoroscopic ablation in the congenital heart disease population. Techniques for procedural safety and the benefits of three-dimensional mapping in the setting of structural heart disease are reviewed.

## Introduction

Pediatric patients with congenital heart disease are at risk for the development of arrhythmias. Many of these arrhythmias are amenable to catheter ablation, but the complexities of these patients’ structural anatomy can negatively affect outcomes. In the modern era of ablation, the use of three-dimensional (3D) mapping has become a staple in the electrophysiology laboratory.^1–4^ In this series, we describe several cases of pediatric patients undergoing nonfluoroscopic ablations in the setting of congenital disease. We also describe how 3D mapping was instrumental to the ablation procedure and techniques to ensure procedural safety when not using fluoroscopy.

## Case presentations

### Case 1

**Figure 1:**
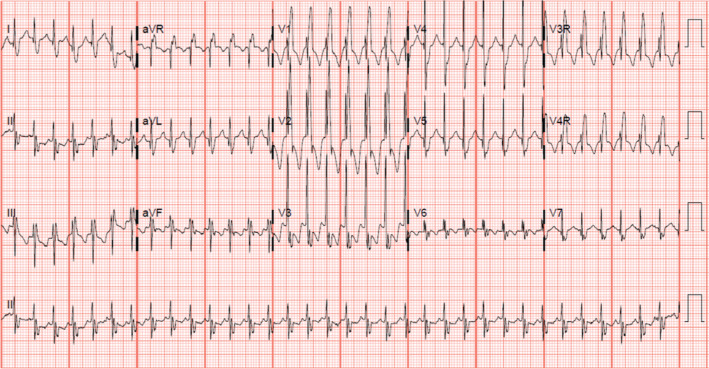
A 15-lead ECG demonstrating intra-atrial reentrant tachycardia with 1:1 conduction and a cycle length of 280 ms.

A six-year-old female with Down syndrome, atrioventricular (AV) septal defect with tetralogy of Fallot, right aortic arch, and left superior vena cava to the coronary sinus presented with arrhythmia. Her initial surgical repair was completed during infancy and subsequent follow-up visits demonstrated a progression of right ventricular outflow tract obstruction and mild to moderate left- and right-sided AV valve regurgitation with right atrial enlargement. At five years of age, she underwent repair of the left and right AV valves, resection of a muscular obstruction of the right ventricular outflow tract, and pulmonary valve replacement with a bioprosthetic valve. Approximately one month after this surgery, the patient began experiencing paroxysmal events of intra-atrial reentrant tachycardia (IART) **(Figure 1)**. She was subsequently admitted and started on sotalol therapy with reasonable control but later demonstrated bradycardia and sinus node dysfunction. Over several months, multiple IART breakthrough episodes occurred associated with decreased energy, vomiting, and poor appetite. Reviewing their options, the family chose to proceed with catheter ablation at eight months after her second surgery.

**Figure 2:**
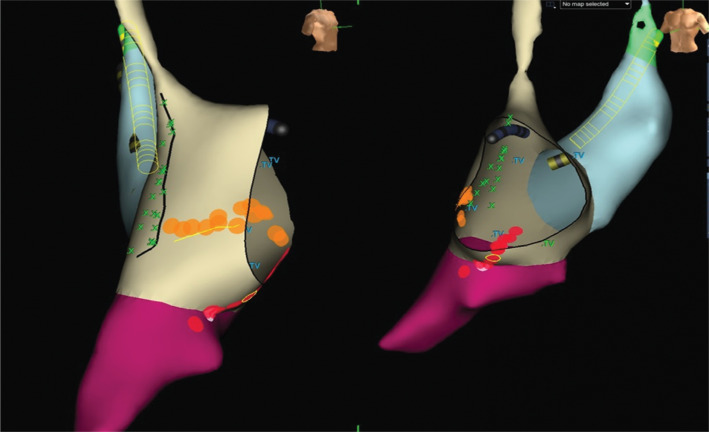
EnSite™ NavX™ geometry of the right atrium demonstrating catheter placement in a dilated coronary sinus secondary to the left superior vena cava. A green X demonstrates an area of diaphragm stimulation demarcated by a black line representing the course of the phrenic nerve. The red and orange dots represent ablation lines created that eliminated the atrial reentrant tachycardia. TV: tricuspid valve.

An EnSite™ Velocity™ 3D mapping system (Abbott Laboratories, Chicago, IL, USA) was used for mapping and ablation. Patches were cut for smaller-sized patients, given this individual’s weight of 15 kg. Geometry of the right atrium including the dilated coronary sinus and left superior vena cava were obtained using a steerable octapolar catheter (Dynamic XT™; Boston Scientific, Natick, MA, USA) **(Figure 2)**. With both decremented atrial pacing and single atrial extra stimuli, IART was reproducibly induced with a cycle length of 280 ms. A 20-pole helix steerable catheter (Inquiry™ Electrophysiology Catheter; Abbott Laboratories, Chicago, IL, USA) was advanced to perform voltage mapping for areas of scar to collect more detailed geometry data and for local activation time mapping with the intent of ablation. Entrainment mapping was performed from the cavotricuspid isthmus, which demonstrated a postpacing interval consistent with involvement in the circuit with a postpacing interval minus tachycardia cycle length of 6 ms. Activation mapping was also performed, demonstrating involvement of the cavotricuspid isthmus and circling around the right-sided AV valve. A bidirectional line of block along the cavotricuspid isthmus was created using a radiofrequency ablation catheter with an 8-mm tip (BLAZER™ II; Boston Scientific, Natick, MA, USA). Postablation testing resulted in the induction of atrial reentrant tachycardia with a cycle length of 300 ms. A late diastolic fractionated potential was noted between an area of scar, presumably from a previous atriotomy incision, and the tricuspid valve annulus at approximately the 8 o’clock position. Entrainment revealed a postpacing interval minus tachycardia cycle length of 4 ms. Given concerns regarding the location of the phrenic nerve, high output pacing was performed along the lateral wall of the right atrium to identify the course of the phrenic nerve. Sites of phrenic nerve stimulus signaled by diaphragm movement were tagged on the 3D mapping system **(Figure 2)**. With the phrenic nerve identified and appearing relatively close to the potential area of ablation, the ablation catheter was exchanged for one with a 4-mm tip. A bidirectional line of block was then created between the atriotomy scar and the tricuspid valve annulus. Postablation IART could not be reinduced. No fluoroscopy was used, the patient had no complications, and no recurrence of IART during follow-up was noted.

### Case 2

**Figure 3:**
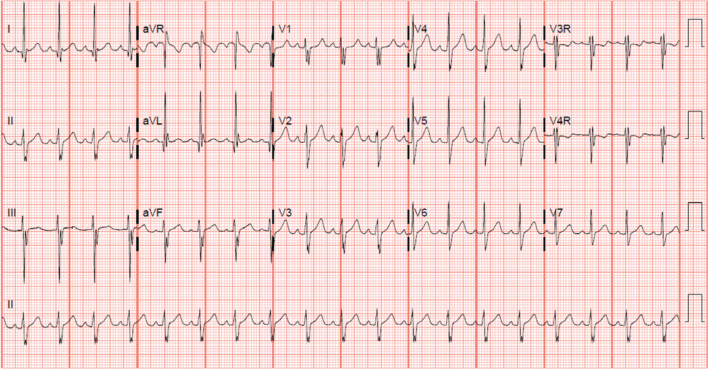
A 15-lead ECG demonstrating sinus rhythm with a left or superior QRS vector suggesting a posteriorly displaced AV node, as is seen in a primum atrial septal defect.

A 12-year-old male presented to the cardiology clinic due to intermittent palpitations. He was seen in the emergency room for an episode of tachycardia, which appeared to be a narrow QRS complex tachycardia at a rate of 220 bpm that resolved with the administration of adenosine. On physical examination, a murmur was noted and a 15-lead electrocardiogram (ECG) demonstrated superior QRS axis **(Figure 3)**. An echocardiogram was obtained and demonstrated a primum atrial septal defect with a common AV valve. Elective surgical repair was recommended with electrophysiology study and ablation prior to intervention to avoid postoperative arrhythmias and the potential creation of an obstruction to ablation.

**Figure 4:**
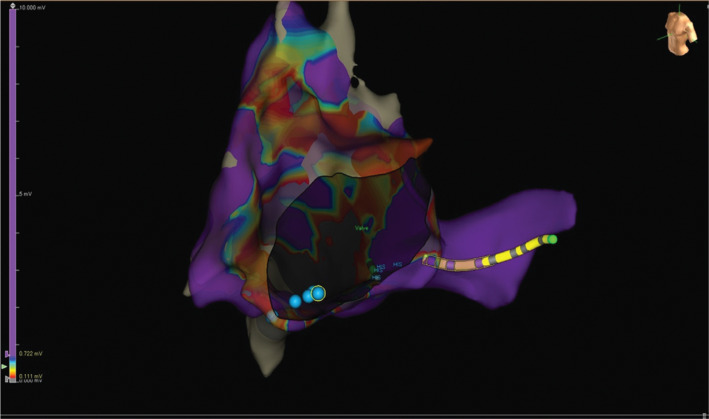
Atrial geometry demonstrating a common AV valve with posterior and inferior displacement of the His bundle. The blue dots represent areas of cryothermal ablation. The yellow catheter is in the coronary sinus.

**Figure 5:**
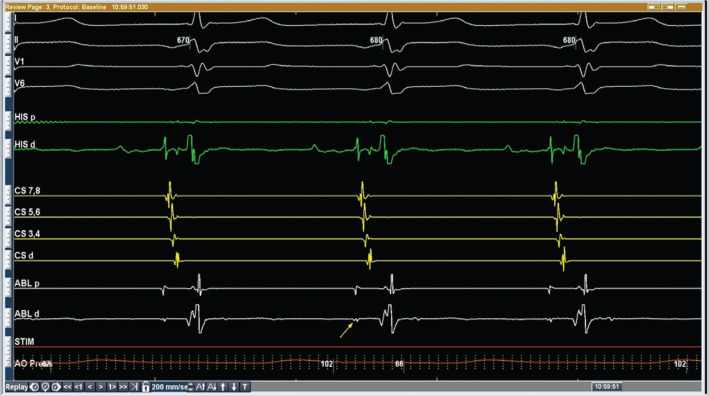
The arrow depicts the slow pathway potential located at approximately the 6 o’clock position of the common AV valve. Top to bottom: four surface ECGs, His catheter (four bipoles), coronary sinus catheter (eight bipoles), cryothermal ablation catheter (four bipoles), stim channel, and arterial pressure waveform.

Using the EnSite™ Precision™ cardiac mapping system (Abbott Laboratories, Chicago, IL, USA), an octapolar steerable catheter was used to create a 3D geometry and sinus rhythm voltage map of the atria without the use of fluoroscopy. Catheters were placed in the coronary sinus, high right atrium, right ventricle apex, and near the His bundle, which was inferiorly displayed as is seen in this type of defect **(Figure 4)**. Adenosine resulted in block in both ventriculoatrial and AV conduction. Atrial burst pacing resulted in reproducible narrow complex tachycardia of a cycle length of 310 ms and a ventriculoatrial interval of less than 10 ms, suggesting typical AV nodal reentry tachycardia. A His-refractory premature ventricular complex was delivered, which did not alter the tachycardia cycle length in the atrium. A voltage map was created as previously described^5^ to identify areas of low voltage and to assist in the generation of a propagation map. The area of collision on the propagation map was noted inferiorly on the common valve annulus, at approximately the 6 o’clock position. Slow pathway electrograms were noted in this location as well **(Figure 5)**. Given the proximity of the His bundle to the slow pathway area and the previously demonstrated higher incidence of AV conduction block in congenital heart disease patients undergoing ablation, we elected to proceed with cryothermal ablation of the slow pathway using a 6-mm-tip cryothermal ablation catheter (Freezor™ Xtra Catheter; Medtronic, Minneapolis, MN, USA) with lesions toward the inferior portion of the common AV valve. After a 30-minute waiting period and using an aggressive stimulation protocol with isoproterenol, there was no inducible tachycardia or evidence of slow pathway function. The patient tolerated the procedure well without complication. At approximately six weeks postablation, the patient was sent to the operating room for repair of his primum atrial septal defect and common AV valve. There were no intraoperative or postoperative arrhythmias noted and the patient continues to be arrhythmia-free to date.

### Case 3

**Figure 6:**
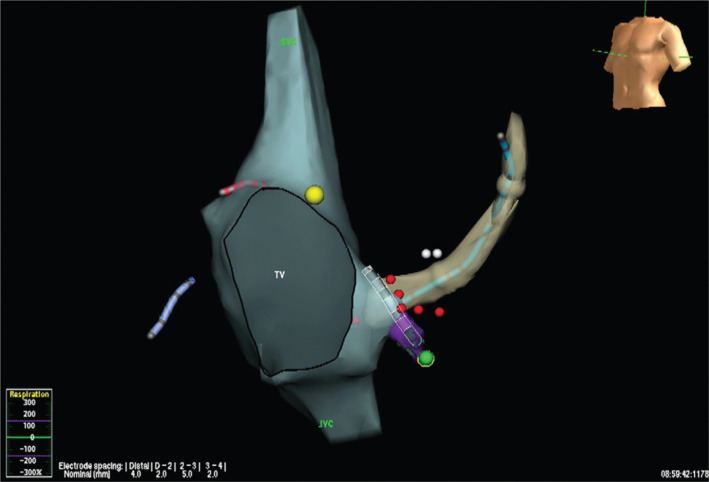
EnSite™ NavX™ atrial geometry demonstrating diverticulum of the coronary sinus (purple). The green dot represents the site of accessory pathway elimination within the coronary sinus diverticulum. IVC: inferior vena cava; SVC: superior vena cava; TV: tricuspid valve.

A 12-year-old patient presented with documented supraventricular tachycardia and Wolff-Parkinson-White syndrome pattern on baseline ECG suggestive of a septal accessory pathway **(Figure 6)**. Electrophysiology study and catheter ablation was performed with the EnSite™ NavX™ 3D mapping system (Abbott Laboratories, Chicago, IL, USA). Catheters were placed in the high right atrium, His bundle, right ventricle, and coronary sinus positions without the use of fluoroscopy. The unique feature of this case was the presence of a coronary sinus diverticulum **(Figure 6)**.

Coronary sinus diverticulum is a rare anatomic anomaly. It is well-known that manifest and concealed accessory pathways can be present in these unusual pouches, which typically do not cause other problems. Venography of the coronary sinus can help to reveal the diverticulum, but it can also be identified solely with 3D mapping systems, maintaining a fluoroless procedure. **Figure 6** shows a 3D geometry of coronary sinus diverticulum in a seven-year-old child with local activation time mapping of the V-delta using minimal interpolation (4 mm) and fluoroless technique.

The ablation catheter could not be advanced further into the diverticulum and the earliest V-delta mapping showed that an accessory pathway was present within this anatomical structure. Although angiographic delineation was not performed, the diverticulum was implied with (1) 3D geometry creation of the structure just within the coronary sinus ostium inferiorly, which is the usual location of coronary sinus diverticulae; (2) the presence of an accessory pathway within this structure; and (3) the inability to advance the catheter further, which provided evidence that the catheter was not in the middle cardiac vein.

## Discussion

Radiation use in the electrophysiology laboratory should adhere to the As Low As Reasonably Achievable (ALARA) principle. Radiation can have significant impacts on both the patients and staff involved in these procedures.^6–8^ Primary operators must exercise good judgement and attempt conservative fluoroscopy use.

The evolution of 3D mapping systems has had significant impacts on the reduction of fluoroscopy use during electrophysiology study and ablation. Benefits include monitoring of catheter movement, identification and anatomic tagging of structures, and display of ablation sites. Performance of nonfluoroscopic procedures in the electrophysiology laboratory is becoming more commonplace.

All cases presented herein used the EnSite™ 3D mapping system (Abbott Laboratories, Chicago, IL, USA), which is an impedance-based system. With vascular access established, a catheter is advanced to the atrium while being tracked by the mapping system. It is advised to use a soft, flexible, and steerable catheter to minimize the risk of vascular injury and maximize manipulation. If the user collects the geometry while advancing the catheter, a map of the vascular anatomy can be established for the placement of other catheters. Advancement of the catheter should occur without any resistance; generally, atrial electrograms noted on the distal pole reflect the inferior vena cava and right atrial junction, which is subsequently anatomically tagged in the system. The catheter is then further advanced with counterclockwise torque toward the superior vena cava. When the distal pole loses electrograms, this site is tagged as the junction of the superior vena cava and right atrial junction. The catheter in this case is then manipulated through the anatomy of the heart, collecting a 3D geometry while tagging specific sites including the AV node, coronary sinus, and tricuspid valve annulus. The placement of additional catheters can be done using the previously made geometry and tagged sites.

Pediatric patients with small size and congenital heart disease can raise significant complexities. However, the benefits of a 3D mapping system can be instrumental in this patient population. Understanding the underlying cardiac anatomy and created surgical anatomy is central to the success of any ablation in congenital heart disease. Using previous operative reports, ECGs, computed tomography, and/or magnetic resonance imaging can be critical in understanding a patient’s anatomy. The use of intracardiac echocardiography or transesophageal echocardiography can also be helpful during the ablation procedure to verify anatomical structures and catheter contact. The ability to recreate the 3D geometry of complex congenital heart disease with catheter manipulation has direct benefits to achieving optimal success in ablation. Manipulation of the 3D image enhances the ablation experience for the provider and can help to demonstrate proximity to various structures that cannot be readily appreciated with two-dimensional fluoroscopy. Voltage mapping allows for the demonstration of areas of suspected scar and the potential for areas of slow conduction. Labeling of structures such as the course of the phrenic nerve or bundle of His can also assist in the prevention of damage to such structures.

The three cases presented here illustrate the safety and efficacy of nonfluoroscopic ablation using 3D mapping in pediatric congenital heart disease with complex arrhythmias. These systems allow the pediatric electrophysiologist to minimize radiation use and heed the ALARA principle. Although fluoroscopy use when necessary should never be discouraged, particularly in the interest of patient safety, 3D mapping systems represent a useful tool to minimize complications and improve outcomes in this patient population.
